# Supplementary material for: Performance of risk prediction models for diabetic foot ulcer: a meta-analysis
Source: PeerJ. 2024 Jul 17;12:e17770. doi: 10.7717/peerj.17770 (PMC11260075; doi:10.7717/peerj.17770)
Supplement: Supplemental Information 1 [file peerj-12-17770-s001.docx]

**The retrieval strategies and retrieval results of each database are shown in Tables 1-3**.

**Table 1** **|** PubMed

| No. | Content | Result |
| --- | --- | --- |
| #1 | Search: "Diabetes Mellitus"[Mesh] Sort by: Most Recent | 515,141 |
| #2 | Search: Diabet*[Title/Abstract] Sort by: Most Recent | 797,585 |
| #3 | Search: ("Diabetes Mellitus"[Mesh]) OR (Diabet*[Title/Abstract]) Sort by: Most Recent | 859,015 |
| #4 | Search: (((Prediction model[Title/Abstract]) OR (Prognostic model[Title/Abstract])) OR (risk prediction[Title/Abstract])) OR (risk prediction model[Title/Abstract]) Sort by: Most Recent | 47,688 |
| #5 | Search: (("Diabetes Mellitus"[Mesh]) OR (Diabet*[Title/Abstract])) AND ((((Prediction model[Title/Abstract]) OR (Prognostic model[Title/Abstract])) OR (risk prediction[Title/Abstract])) OR (risk prediction model[Title/Abstract])) Sort by: Most Recent | 3,597 |

**Table 2 |** Embase

| No. | Content | Result |
| --- | --- | --- |
| #1 | 'diabetes mellitus'/mj | 265,847 |
| #2 | 'diabetes mellitus':ab,ti OR diabet*:ab,ti | 1,217,118 |
| #3 | #1 OR #2 | 1,245,537 |
| #4 | 'prediction model':ab,ti OR 'prognostic model':ab,ti OR 'risk prediction':ab,ti OR 'risk prediction model':ab,ti | 60,894 |
| #5 | #3 AND #4 | 5,382 |

**Table 3 |** Cochran Library

| No. | Content | Result |
| --- | --- | --- |
| #1 | MeSH descriptor: [Diabetes Mellitus] this term only | 17,403 |
| #2 | (diabetes mellitus):ti,ab,kw OR (diabet*):ti,ab,kw | 118,698 |
| #3 | #1 OR #2 | 118,698 |
| #4 | (prediction model):ti,ab,kw OR (prognostic model):ti,ab,kw OR (risk prediction):ti,ab,kw OR (risk prediction model):ti,ab,kw | 49,382 |
| #5 | #3 AND #4 | 4,583 |
