# Supplementary material for: Performance of risk prediction models for diabetic foot ulcer: a meta-analysis
Source: PeerJ. 2024 Jul 17;12:e17770. doi: 10.7717/peerj.17770 (PMC11260075; doi:10.7717/peerj.17770)
Supplement: Supplemental Information 2 [file peerj-12-17770-s002.docx]

**1.Prediction models for diabetic foot ulcer in patients are useful for identifying the potential complication, and enabling timely interventions. While the quality and applicability of these models in clinical practice and future research remain unknown. To systematically review published studies on risk prediction models for diabetic foot ulcer in patients and discuss and summary their methodologies and predictors.**

**﻿**

**2.While the prediction model has continued to advance and technology improve, many predictive models in widespread use, when critically evaluated, have been found to neither adhere to reporting standards nor perform as well as expected. DFU risk prediction models generally exhibit good overall predictive accuracy. Nonetheless, there's a notable risk of bias during their development and validation phases. It's vital to improve the calibration performance of existing models, ensuring their suitability for the general population. In future research, priority should be given to assessing model applicability and closely following the PROBAST checklist to enhance clinical relevance.**

**Figure A**


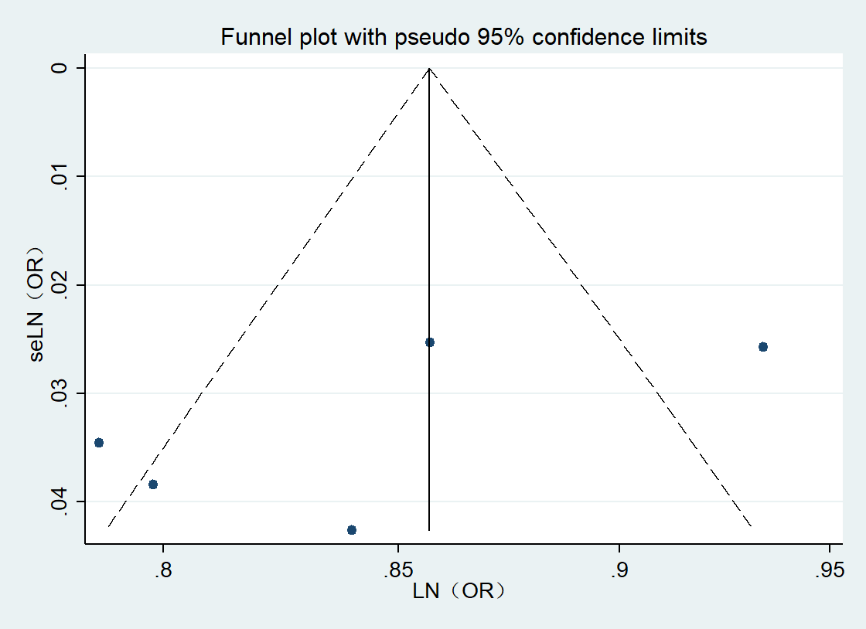

**Figure B**
